# Raltitrexed regulates proliferation and apoptosis of HGC-27 cells by upregulating RSK4

**DOI:** 10.1186/s40360-022-00605-2

**Published:** 2022-08-28

**Authors:** Cong Hu, Xinhua Chen, Xu Lin, Jun Dai, Jiang Yu

**Affiliations:** 1 Department of General Surgery, Nanfang Hospital, Southern Medical University, No. 1929, Guangzhou Avenue North, Baiyun District, Guangzhou, 510515 China; 2 Department of General Surgery, Zhuhai People’s Hospital, Zhuhai Hospital Affiliated With Jinan University, Zhuhai, 519000 China

**Keywords:** Thymidylate synthase inhibitor, Gastric cancer, RSK4, Raltitrexed

## Abstract

**Background:**

Raltitrexed is a specific inhibitor of thymidylate synthase and a potential chemotherapeutic agent for the treatment of advanced gastric cancer. In this study, we investigated the effect of raltitrexed on the proliferation of HGC-27 human gastric cancer cells and its potential underlying molecular mechanism(s).

**Methods:**

RT-qPCR and western blotting were used to quantify RSK4 levels. Colony formation and flow cytometry assays were used to assess HGC-27 cell proliferation, cell cycle progression, mitochondrial membrane potential, and apoptosis. The expression of cell cycle and apoptosis markers were determined by western blotting.

**Results:**

Our results demonstrate that raltitrexed upregulated RSK4 mRNA and protein levels in HGC-27 cells. Moreover, raltitrexed significantly inhibited tumor cell colony formation, arrested the cell cycle, decreased the mitochondrial membrane potential, and induced apoptosis. We observed that raltitrexed was capable of upregulating the expression of Bax, cyclin A1, and CDK3, and downregulating the expression of Bcl-2 and cleaved caspase-3. Importantly, siRNA-mediated RSK4 knockdown significantly reduced the inhibitory effect of raltitrexed on cell proliferation and its promotion of cell apoptosis. Moreover, silencing of RSK4 inhibited the raltitrexed-induced upregulation of cytochrome C. In addition, the changes in molecular markers related to the cell cycle and apoptosis induced by raltitrexed were reduced upon RSK4 depletion.

**Conclusion:**

Our study shows that RSK4 is a key target of raltitrexed in the regulation of gastric cancer cell proliferation, cell cycle progression, and apoptosis.

**Supplementary Information:**

The online version contains supplementary material available at 10.1186/s40360-022-00605-2.

## Introduction

Gastric cancer is the fifth most common malignant tumor worldwide [1]. Although its incidence has decreased during the past century, gastric cancer remains the third most deadly cancer in East Asia [1–4]. Previous studies have shown that the 5-year survival rate of gastric cancer can be significantly increased from 49.6% to 55.3% by adjuvant chemotherapy [5]. Additionally, according to preliminary clinical observations, patients diagnosed with advanced gastric cancer can gain survival advantages by combined treatment with raltitrexed and other medicines (e.g., paclitaxel or docetaxel) compared with raltitrexed only [5].

Raltitrexed, also known as ZD1694 or Tomudex®, is a specific thymidylate synthase (TS) inhibitor that can reduce the proliferation of tumor cells by arresting the cell cycle in the G0/G1 phase and by inducing tumor cell apoptosis through the mitochondrial pathway [5, 6]. Clinical data indicate that the anti-cancer activity of raltitrexed can lead to improved treatment outcomes in various types of cancer, including colorectal cancer, malignant mesothelioma, head and neck cancer, liver cancer, and stomach cancer [7]. The most common adverse events associated with raltitrexed are neutropenia, diarrhea, and elevated liver enzymes [8]. Compared with 5-FU, raltitrexed has better hematological and gastrointestinal tolerability, and because it does not cause accumulation of related metabolites, it exhibits reduced cardiotoxicity in patients [5, 9]. Raltitrexed is associated with a significantly lower incidence of severe leukopenia and mucositis compared with 5FU plus leucovorin [9]. Currently, raltitrexed has been approved for use in the treatment of advanced colorectal cancer [5, 7]. Although TS is the main target in raltitrexed treatment, it remains unknown whether other possible targets are involved in its tumor-suppressing activity. Hence, a better understanding of the molecular mechanism(s) of raltitrexed is needed, which would be conducive to its clinical promotion and application.

RSK4, also known as RPS6KA6, belongs to the p90 ribosomal protein S6 kinase (RSK) family, and it plays an essential role in cell proliferation, migration, and invasion [1, 10]. RSK is considered to be a marker for assessing patient prognosis since it significantly inhibits cell proliferation, migration, and invasion in colon, breast, and gastric cancer [11–13]. Moreover, overexpression of RSK4 can reverse the drug resistance of human breast cancer cells to doxorubicin by activating the PI3K/Akt signaling pathway [14]. Our previous data have indicated that RSK4 is upregulated upon raltitrexed treatment. To provide a more theoretical basis for its further clinical application, in this study we investigated whether the anti-tumor effect of raltitrexed is related to RSK4 upregulation.

## Methods

### Cell culture

The gastric cancer cell line HGC-27 was purchased from Xiamen Immocell Biotechnology Co., Ltd. (Cat. No.: IM-H085; China, Xiamen) and maintained in high-glucose Dulbecco’s Modified Eagle’s Medium (DMEM; Gibco; Cat. No.:11965–0092; China, Shanghai) supplemented with 100 U/mL penicillin/streptomycin (Gibco; Cat. No. 15070–063;), and 10% fetal bovine serum (FBS; Gibco; Cat. No.: S1860-500).

### Experimental models

Small interfering RNA targeting RSK4 (siRSK4) was designed based on the human RSK4 sequence (GenBank accession number: NM_014496.5). siRSK4 sense: 5′-UUUACCUUGUUACGGAUUUAA-3′, and siRSK4 antisense: 5′-UUAAAUCCGUAACAAGGUAAA-3′. HGC-27 cells underwent four types of treatment: dimethyl sulfoxide (DMSO) + siNC, DMSO + siRSK4, raltitrexed + siNC, and raltitrexed + siRSK4. In the first two groups, the cells were transfected with negative control siNC or siRSK4, respectively, and then treated with 0.1% DMSO for 48 h. In contrast, cells in the last two groups were transfected with negative control siNC or siRSK4, respectively, and subsequently treated with 0.5 µg/mL Raltitrexed (Selleck; Cat. No.: S1192; China, Shanghai) for 48 h. The transfections were performed using Lipofectamine™ 3000 reagent (Invitrogen; Cat. No.: L3000001; China, Shanghai) following the standard manufacturer’s recommendations.

### Colony formation

HGC-27 cells were seeded at a density of 500 cells/well in 6-well plates at 48 h post-transfection. After subculturing for two weeks at 37 °C, the cells were fixed with 4% paraformaldehyde (PFA) for 10 min and subsequently stained with 0.5% crystal violet for 30 min at room temperature. The stained cells were imaged and counted after several washes.

### Cell cycle analysis

Cells were harvested and stained with 20 μg/mL propidium iodide (PI) in the dark at 28 °C for 30 min. The cells were then washed with phosphate-buffered saline (PBS) before being analyzed using a NovoCyte® flow cytometer (Agilent, China, Hangzhou).

### Cell apoptosis analysis

Trypsin (0.25%) without EDTA was used to harvest both floating and adherent cells. The cells were then washed once with PBS and stained with annexin V-FITC according to the manufacturer’s instructions (Beyotime; Cat. No.: C1062S; China, Shanghai). Finally, the fluorescent signals were detected and analyzed using a NovoCyte® flow cytometer (Agilent).

### Western blotting

Total protein was extracted using cold RIPA buffer (Beyotime; Cat. no.: P0013C), and bicinchoninic acid (BCA) solution (Beyotime; Cat. no.: P0012S) was used to determine the protein concentration following the manufacturer’s protocol. The proteins were then subjected to 10% sodium dodecyl-sulfate polyacrylamide gel electrophoresis (SDS-PAGE) and transferred onto polyvinylidene difluoride (PVDF) membranes (Millipore; Cat. No. IPFL00010, China, Shanghai). The membranes containing the electrophoresed proteins were blocked with 5% non-fat milk at 25 °C for 2 h and then incubated with the appropriate primary antibodies against RSK4 (Abcam; Cat. No.: ab76117; 1:1000 dilution; China, Shanghai), cleaved caspase-3 (Cell Signaling Technology; Cat. No.9664; 1:1000 dilution; China, Shanghai), Bax (Cell Signaling Technology; Cat. No.89477; 1:1000 dilution), Bcl-2 (Cell Signaling Technology; Cat. No.15071; 1:1000 dilution), cyclin A1 (Abcam; Cat. No.: ab53699), CDK2 (ProteinTech; Cat. No.: 10122–1-AP; China, Wuhan) and GAPDH (ProteinTech; Cat. No.60004–1-Ig) at 25 °C for 2 h. After three washes with Tris–HCl buffer, the membranes were probed with horseradish peroxidase (HRP)-conjugated secondary antibodies against mouse IgG or rabbit IgG at 25 °C for 1 h. The membranes were washed several times before signal detection.

### Mitochondrial membrane potential analysis

At 48 h post-transfection, measurement of the mitochondrial membrane potential was performed using a JC-1 Mitochondrial Membrane Potential Kit (MedChemExpress; Cat. no.: HY-K0601; China, Shanghai) according to the manufacturer’s suggestions and a previous description [15]. The cells were then analyzed by flow cytometry (NovoCyte® FACS). Green (JC-1 monomer) and red (JC-1 aggregates) fluorescence were detected within the FITC-channel (Ex: 488 nm/Em: 519 nm) and PE-channel (Ex: 488 nm/Em: 578 nm), respectively. The mean fluorescence intensity (MFI) was measured and the green/red MFI ratio was then calculated.

### RT-qPCR

Total RNA was isolated using an RNA isolation kit (Sigma; Cat. No.:83913-1EA; China, Shanghai), and the resulting RNA was reverse transcribed using a reverse transcription kit (Vazyme; Cat. No.: R101-01/02; Nanjing, China). Target genes were amplified using a ChamQ SYBR® qPCR Master Mix Kit (Vazyme; Cat. No.: Q331-02), and the signals were detected using an iQ™5 real-time PCR system (Bio-Rad Laboratories, Hercules, CA, USA). The expression levels of the various target genes were normalized to the level of 18S rRNA using the standard 2^−ΔΔCq^ formula. The primers used in this study were designed based on the human RSK4 sequence (GenBank Accession Number: NM_014496.5) and 18S rRNA (GenBank Accession Number: NR_145820.1) and were as follows: 18S rRNA Forward: 5′-CGACGACCCATTCGAACGTCT-3′; 18S rRNA Reverse: 5′-CTCTCCGGAATCGAACCCTGA-3′; RSK4 Forward: 5′-CCTCCTTTCAAACCTGCTTCTGG-3′; RSK4 Reverse: 5′-GCTGATGAGCATTTGCACTGGC-3′.

### Statistical analysis

Statistical analyses were performed using SPSS software (Version 22.0). Statistical significance was calculated using the unpaired Student’s *t*-test for comparison between two groups. One-way ANOVA was performed to compare differences among multiple groups. GraphPad Prism software (version 8.2.1) was used to generate figures, and the data are presented as means ± s.d. *P*-values less than 0.05 were considered statistically significant. All experiments were performed independently at least three times.

## Results

### Raltitrexed promotes the expression of RSK4

**Fig. 1:**
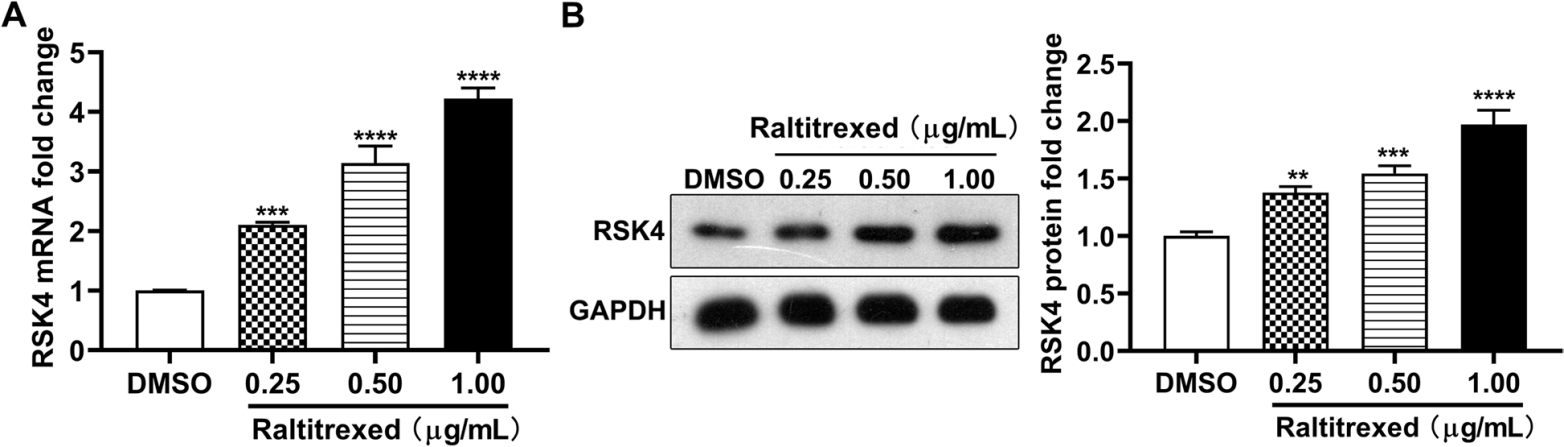
The effect of raltitrexed on *RSK4* expression. **A** The mRNA level of *RSK4* was determined by fluorescence-based quantitative PCR; **B** Representative western blot images of RSK4 protein levels (left) and quantification histograms of the RSK4 protein levels (right). ** *P* < 0.01, *** *P* < 0.001, **** *P* < 0.0001

As our previous study [1] found that the mRNA and protein levels of RSK4 in HGC-27 cells were higher than the levels in other gastric cancer cell lines (SGC-7901 and MGC-803), HGC-27 cells were used in this study. We started our study by exploring whether RSK4 levels could be regulated by raltitrexed in the gastric cell line HGC-27. RT-PCR quantification revealed that the expression of *RSK4* was significantly upregulated in HGC-27 cells upon treatment with raltitrexed for 48 h in a dose-dependent manner compared with the DMSO control group (Fig. 1A). Western blot analysis confirmed that the protein levels of RSK4 were also upregulated in response to raltitrexed treatment (Fig. 1B). Collectively, our results indicate that raltitrexed can promote RSK4 expression at both the mRNA and protein levels.

### Depletion of RSK4 reduces the inhibitory effect of raltitrexed on cell proliferation

**Fig. 2:**
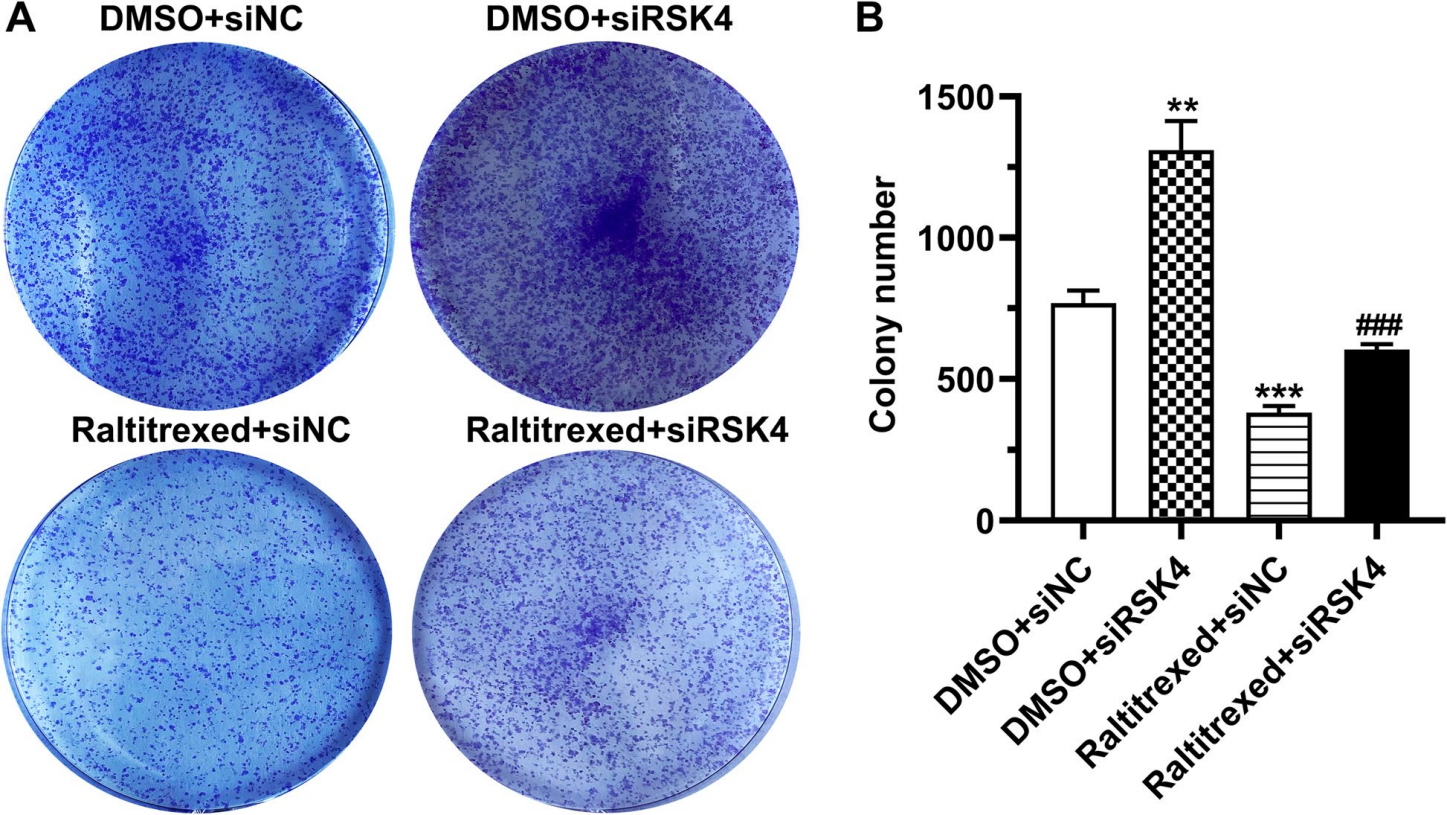
The effect of raltitrexed on cell colony formation. **A** Representative images of the cell colony formation assay; **B** Quantification histograms of the colony numbers. ** *P* < 0.01, *** *P* < 0.001 vs. DMSO + siNC; ### *P* < 0.001 vs. raltitrexed + siNC

**Fig. 3:**
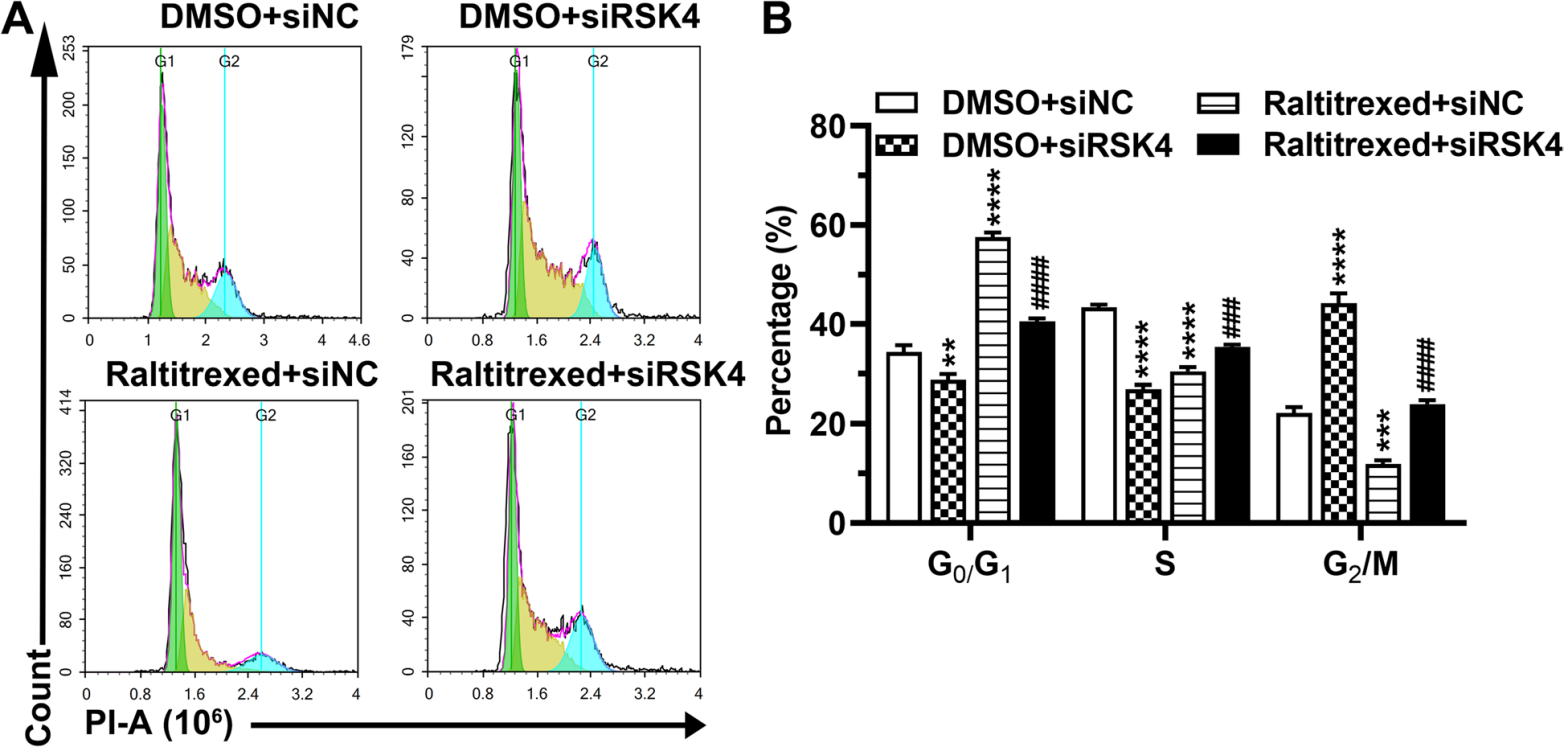
The effect of raltitrexed on the cell cycle. **A** Representative images of the cell cycle assay; **B** Quantification histograms of the proportion of cells in the various cell cycle phases. ** *P* < 0.01, *** *P* < 0.001, **** *P* < 0.0001 vs. DMSO + siNC; ### *P* < 0.001, #### *P* < 0.0001 vs. raltitrexed + siNC

To investigate whether RSK4 is involved in the biological effects exerted by raltitrexed, RSK4 was depleted in HGC-27 cells using a specific siRNA. In the absence of the siRNA against RSK4, treatment of HGC-27 cells with raltitrexed significantly inhibited the colony formation by the cells (Fig. 2A, B). In addition, we observed that in the DMSO control group, RSK4 knockdown resulted in an increase in the number of cell colonies, indicating a cell growth-suppressive role for RSK4 (Fig. 2A, B). However, knockdown of RSK4 almost completely eliminated the attenuation of cell proliferation induced by raltitrexed (Fig. 2A, B). Next, we analyzed whether the effects of RSK4 on the raltitrexed-mediated inhibition of proliferation correlated with alteration of the cell cycle. The percentage of cells in each phase of the cell cycle was quantified by flow cytometry analysis of PI staining. As expected, we found that RSK4 depletion enhanced, whereas raltitrexed treatment reduced cell cycle progression (Fig. 3A, B). Notably, knockdown of RSK4 dramatically reversed the suppressive effect of raltitrexed on cell cycle progression (Fig. 3A, B). Taken together, our results show that RSK4 is a potential downstream target of raltitrexed-induced inhibition of gastric cancer cell proliferation.

### RSK4 knockdown prevents raltitrexed-induced cell apoptosis

**Fig. 4:**
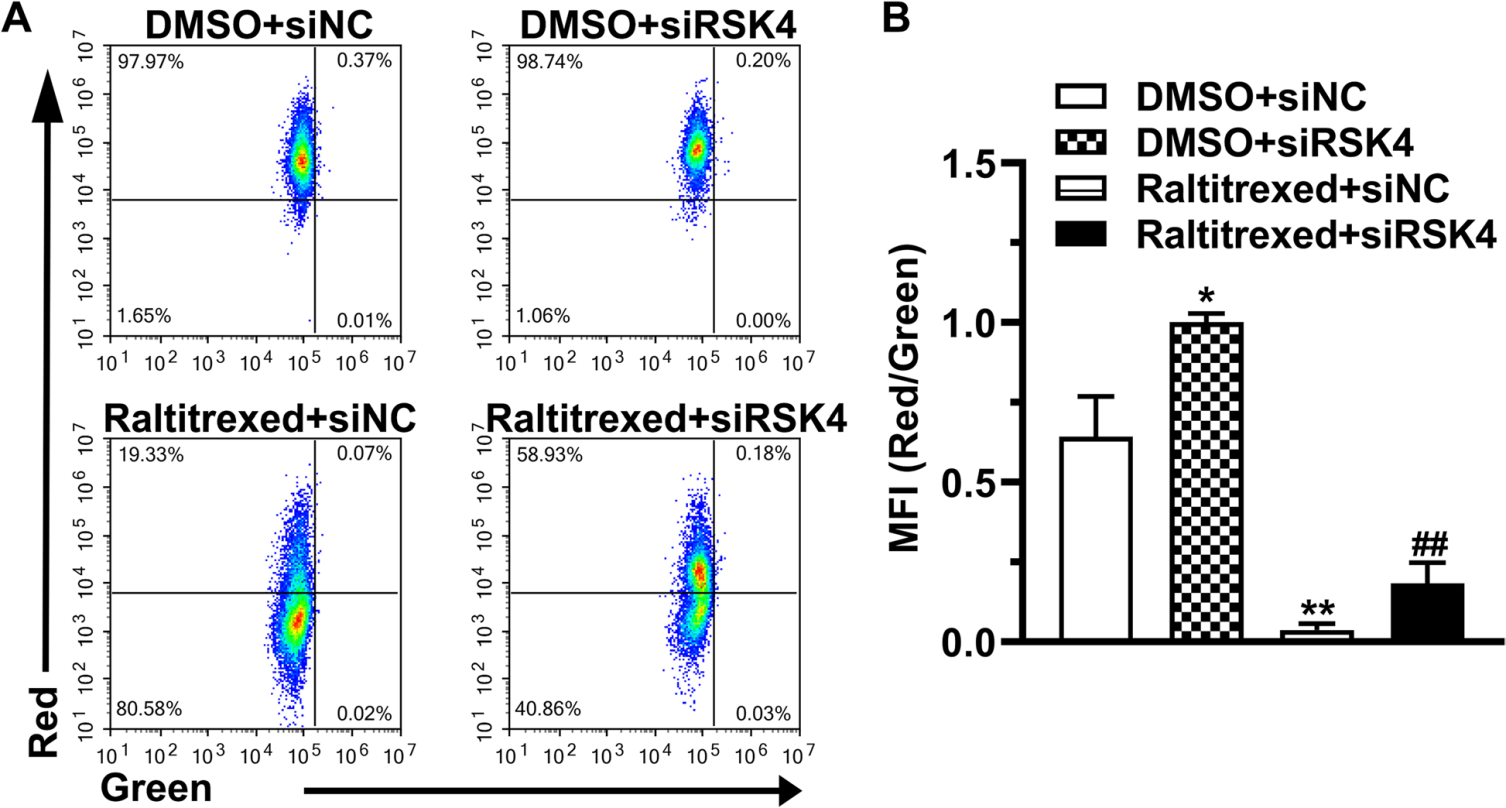
The effect of raltitrexed on the mitochondrial membrane potential. **A** Representative images of the mitochondrial membrane potential assay; **B** Quantification histograms of the red-green fluorescence ratio. MFI: mean fluorescence intensity. * *P* < 0.05, ** *P* < 0.01 vs. DMSO + siNC; ## *P* < 0.01 vs. raltitrexed + siNC

**Fig. 5:**
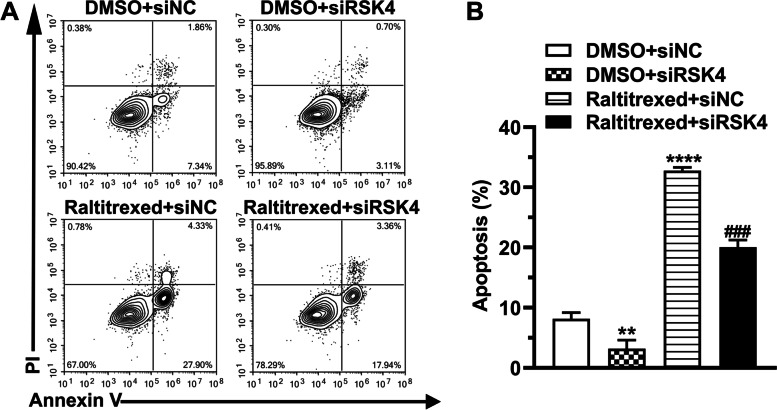
The effect of raltitrexed on apoptosis. **A** Representative images of the apoptosis assay; **B** Quantification histograms of the percentage of apoptotic cells. ** *P* < 0.01, **** *P* < 0.0001 vs. DMSO + siNC; ### *P* < 0.001 vs. raltitrexed + siNC

We investigated the function of RSK4 in raltitrexed-induced cell apoptosis by assaying mitochondrial membrane depolarization using JC-1. Flow cytometry revealed that the ratio of the mean red versus green fluorescence intensity (MFI), which is an indicator of the mitochondrial membrane potential, decreased when cells were stimulated with raltitrexed, whereas it increased when they were transfected with siRNA against RSK4 (Fig. 4A, B). Notably, depletion of RSK4 significantly attenuated the suppression of mitochondrial membrane potential by raltitrexed treatment (Fig. 4A, B). Additionally, we measured the proportion of apoptotic cells by PI-Annexin V double-staining. Similar to the results of the JC-1 assay, knockdown of RSK4 dramatically reduced the apoptosis triggered by raltitrexed treatment of HGC-27 cells (Fig. 5A, B). In summary, in addition to its effects on cell proliferation, RSK4 was shown to be a key molecule in raltitrexed-induced apoptosis.

### RSK4 depletion reduces raltitrexed-induced changes in cell cycle and apoptosis markers

**Fig. 6:**
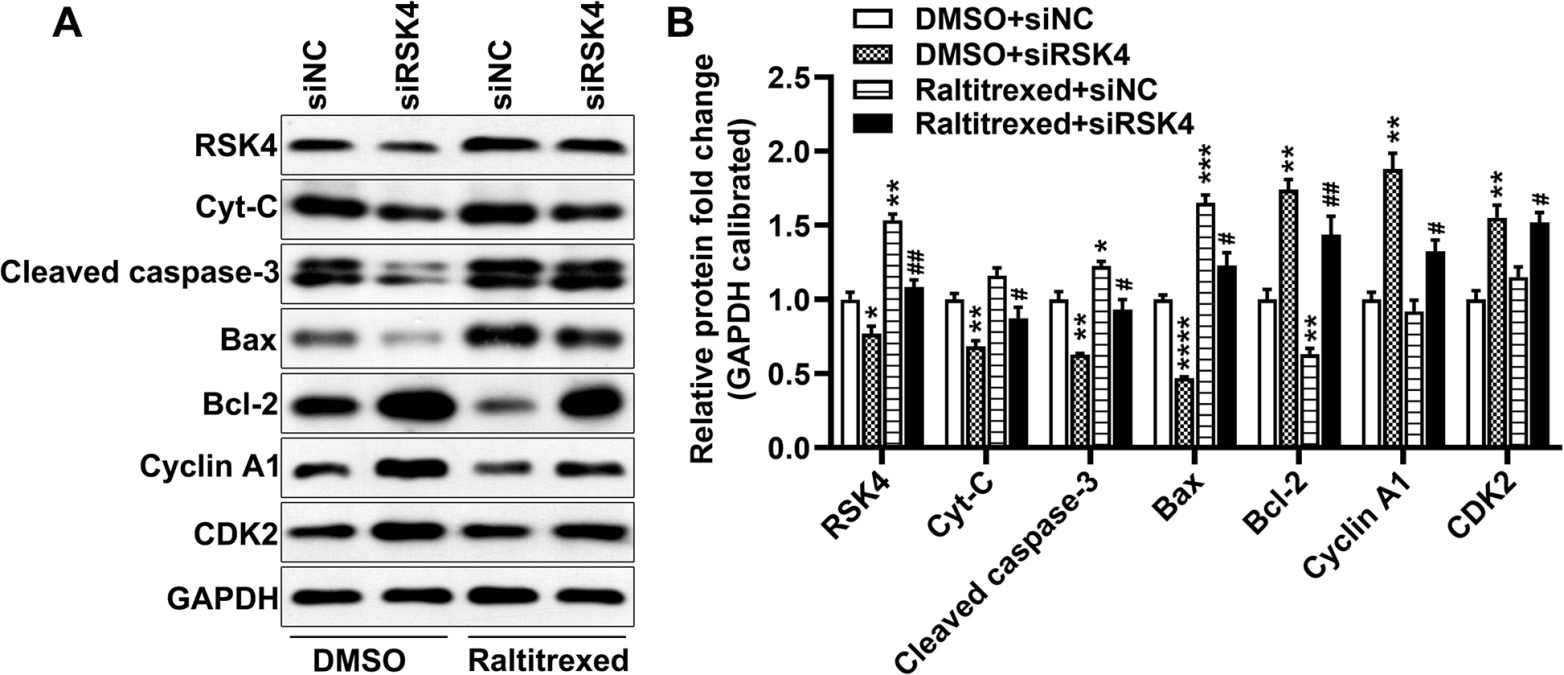
RSK4 knockdown reduced raltitrexed effects on the levels of cell cycle- and apoptosis-related molecules. **A** Representative images of the western blot assay; **B** Quantification histograms of the protein levels. Cyt-C: cytochrome C. * *P* < 0.05, ** *P* < 0.01 vs. DMSO + siNC; # *P* < 0.05, ## *P* < 0.01 vs. Raltitrexed + siNC

Finally, we examined the function of RSK4 in regulating cell cycle arrest and apoptosis induced by raltitrexed at the molecular level. The protein levels of cell cycle and apoptosis molecular markers were determined by western blotting. This showed that RSK4 knockdown significantly increased the basal protein levels of the apoptosis resistance marker Bcl-2 and cell cycle markers cyclin A1 and CDK2, and decreased the protein levels of cytochrome C, cleaved caspase-3, and Bax (Fig. 6A, B). Raltitrexed, on the other hand, reduced Bcl-2 levels and increased RSK4, cleaved caspase-3, and Bax protein levels in siNC-transfected cells (Fig. 6A, B). Importantly, depletion of RSK4 partially blocked the raltitrexed-induced changes in cleaved caspase-3, Bcl-2, Bax, cyclin A1, cytochrome C, and CDK2 (Fig. 6A, B). In conclusion, RSK4 mediates the effect of raltitrexed on the cell cycle and apoptosis of gastric cancer cells.

## Discussion

Gastric cancer is one of the most common cancers worldwide and its etiology is associated with numerous factors [16, 17]. Early-stage gastric cancer is primarily treated by surgery, whereas chemotherapy is the main treatment for middle- and late-stage gastric cancer [5]. Currently, 5-FU is widely used to treat gastric cancer, but the occurrence of drug resistance and side effects has been frequently reported in many patients. Hence, there is an urgent need to develop alternative drugs with less toxicity [5]. The chemotherapeutic agent raltitrexed can specifically inhibit the activity of TS with lower cardiac toxicity [18, 19], making it a potential replacement for 5-FU [20–22]. Clinical data have shown that patients diagnosed with advanced gastric cancer benefit from raltitrexed treatment [18, 19]. However, the underlying mechanisms of raltitrexed-mediated anticancer effects remain unknown.

Indeed, it has been shown that raltitrexed can lead to apoptosis, reduction of mitochondrial membrane potential, and G0/G1 cell cycle arrest in SGC7901 cells [5]. In line with these results, our data demonstrated that raltitrexed significantly suppressed the survival of HGC-27 cells, arrested the cell cycle in G0/G1 phase, and decreased the mitochondrial membrane potential. Moreover, raltitrexed promoted apoptosis by downregulating Bcl-2 expression, upregulating Bax levels, and increasing cleaved caspase-3. Interestingly, these raltitrexed-mediated effects were inhibited by reduction of the RSK4 level. In addition, other studies have indicated that raltitrexed leads to S phase accumulation in SW620 colorectal cancer cells, suggesting that the cell cycle arrest by raltitrexed is cell type-dependent [23].

Our study also reported that raltitrexed upregulated the mRNA and protein levels of RSK4 in HGC-27 cells, indicating that it might affect *RSK4* gene transcription and, thereby, lead to increased RSK4 protein levels. However, the underlying mechanism requires further investigation. Studies have shown that RSK4 levels are suppressed in a variety of cancers, including breast, colorectal, and gastric cancer, and overexpression of RSK4 potentiates anti-tumor effects [1, 13, 24]. Consistent with these results, our data in HGC-27 cells showed that reduction of RSK4 expression dramatically increased colony formation, promoted cell cycle progression, and inhibited cell apoptosis. However, RSK4 is highly expressed in lung cancers [25]. Moreover, ectopic expression of RSK4 leads to enhanced cell migration and invasion in clear cell renal cell carcinoma cells [26]. Taken together, RSK4 appears to play diverse roles in different cancers owing to its variety and complexity.

Additionally, another study showed that the levels of Bcl-2, cyclin A1, and CDK2 were downregulated, and Bax expression was upregulated by overexpression of RSK4 in gastric cancer cells [11], which is consistent with the results of our study. Furthermore, cyclin A1 and CDK2 are the two key regulatory factors involved in the G1 to S phase transition, and Bax/Bcl-2 plays more essential roles in cell apoptosis compared to the activation of caspase-3 [6, 27–29]. Our data demonstrated that depletion of RSK4 in raltitrexed-treated HGC-27 cells reduced the activation of caspase-3 and expression of Bax, whereas Bcl-2, cyclin A1, and CDK2 levels were enhanced, suggesting that raltitrexed-mediated cell cycle arrest and cell apoptosis occur via upregulation of RSK4 expression.

Apoptosis is a spontaneous process of programmed cell death. There are three main signal transduction pathways involved in this process: mitochondrial, death receptor, and endoplasmic reticulum signal transduction pathways [5]. Moreover, integration and amplification of apoptosis signals usually occur in mitochondria [30]. In this study, we showed that raltitrexed significantly decreased the mitochondrial membrane potential of HGC-27 cells, and silencing RSK4 inhibited raltitrexed-induced upregulation of cytochrome C, indicating that cell apoptosis induced by raltitrexed is mediated through the mitochondrial pathway. Mitochondria are involved in various cell apoptosis processes, such as reduction of the mitochondrial membrane potential and release of ROS and other cell apoptosis-related proteins [31–33]. Loss of the mitochondrial membrane potential is an early specific event in the mitochondria-mediated apoptosis pathway [5].

Raltitrexed is an inhibitor of TS, which is the rate-limiting enzyme in pyrimidine deoxynucleotide biosynthesis, and inhibition of TS inhibits the cell cycle [34]. While silencing of RSK4 accelerates cell cycle progression, overexpression of RSK4 blocks the cell cycle [1, 11]. Whether TS is related to the effects of RSK4 on the cell cycle remains to be explored, which is a limitation of this study.

In conclusion, our data illustrate that raltitrexed inhibits the growth of HGC-27 cells and regulates the expression of Bax, cytochrome C, Bcl-2, cyclin A1, CDK2, and cleaved caspase-3 by upregulating RSK4 levels, leading to G0/G1 cell cycle arrest, reduction of the mitochondrial membrane potential, and cell apoptosis. However, further in vivo research and clinical investigations are needed to elucidate the fundamental basis for raltitrexed treatment.

## Supplementary Information

**Additional file 1.** Original images. **Figure 1B.** Raltitrexed upregulated the protein level of RSK4. **Figure 6A.** Raltitrexed regulated expressions ofcell cycle- and apoptosis-related by increasing RSK4. 

## Data Availability

The human RSK4 sequence (GenBank accession number: NM_014496.5) and 18S rRNA (GenBank accession number: NR_145820.1) were obtained from GenBank (https://www.ncbi.nlm.nih.gov/genbank/). Other datasets used and/or analyzed during the current study are available from the corresponding author upon reasonable request.
