# Supplementary material for: Raltitrexed regulates proliferation and apoptosis of HGC-27 cells by upregulating RSK4
Source: BMC Pharmacol Toxicol. 2022 Aug 28;23:65. doi: 10.1186/s40360-022-00605-2 (PMC9420250; doi:10.1186/s40360-022-00605-2)

original images

Figure 1B Raltitrexed upregulated the protein level of RSK4.

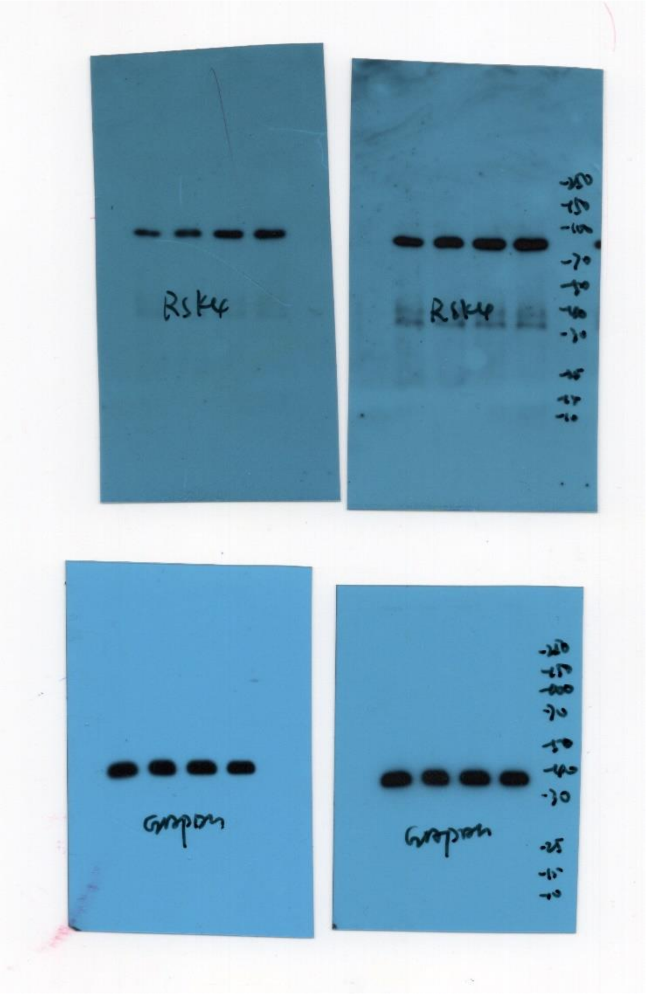

Figure 6A Raltitrexed regulated expressions of cell cycle- and apoptosis-related by increasing RSK4.

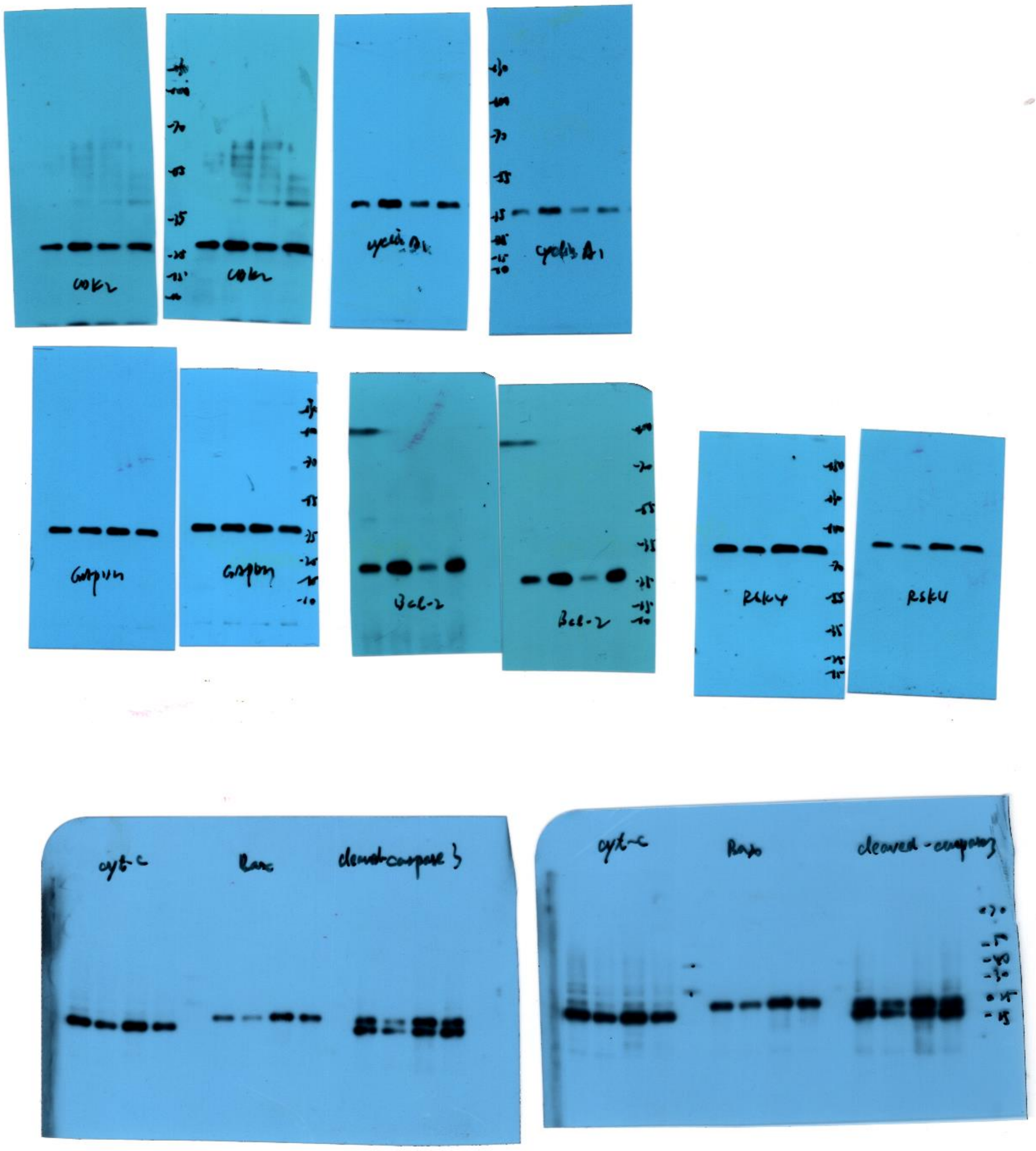

Supplement: Supplementary file 1 — Additional file 1. Original images. Figure 1B. Raltitrexed upregulated the protein level of RSK4. Figure 6A. Raltitrexed regulated expressions ofcell cycle- and apoptosis-related by increasing RSK4. [file 40360_2022_605_MOESM1_ESM.pdf]
